# Pan-genome analyses of 24 *Shewanella* strains re-emphasize the diversification of their functions yet evolutionary dynamics of metal-reducing pathway

**DOI:** 10.1186/s13068-018-1201-1

**Published:** 2018-07-17

**Authors:** Chaofang Zhong, Maozhen Han, Shaojun Yu, Pengshuo Yang, Hongjun Li, Kang Ning

**Affiliations:** Key Laboratory of Molecular Biophysics of the Ministry of Education, Hubei Key Laboratory of Bioinformatics and Molecular-imaging, Department of Bioinformatics and Systems Biology, College of Life Science and Technology, Huazhong University of Science and Technology, 1037 Luoyu Road, Wuhan, 430074 Hubei China

**Keywords:** Pan-genome, *Shewanella*, Metal-reducing, Evolution

## Abstract

**Background:**

*Shewanella* strains are important dissimilatory metal-reducing bacteria which are widely distributed in diverse habitats. Despite efforts to genomically characterize *Shewanella*, knowledge of the molecular components, functional information and evolutionary patterns remain lacking, especially for their compatibility in the metal-reducing pathway. The increasing number of genome sequences of *Shewanella* strains offers a basis for pan-genome studies.

**Results:**

A comparative pan-genome analysis was conducted to study genomic diversity and evolutionary relationships among 24 *Shewanella* strains. Results revealed an open pan-genome of 13,406 non-redundant genes and a core-genome of 1878 non-redundant genes. Selective pressure acted on the invariant members of core genome, in which purifying selection drove evolution in the housekeeping mechanisms. *Shewanella* strains exhibited extensive genome variability, with high levels of gene gain and loss during the evolution, which affected variable gene sets and facilitated the rapid evolution. Additionally, genes related to metal reduction were diversely distributed in *Shewanella* strains and evolved under purifying selection, which highlighted the basic conserved functionality and specificity of respiratory systems.

**Conclusions:**

The diversity of genes present in the accessory and specific genomes of *Shewanella* strains indicates that each strain uses different strategies to adapt to diverse environments. Horizontal gene transfer is an important evolutionary force in shaping *Shewanella* genomes. Purifying selection plays an important role in the stability of the core-genome and also drives evolution in *mtr*–*omc* cluster of different *Shewanella* strains.

**Electronic supplementary material:**

The online version of this article (10.1186/s13068-018-1201-1) contains supplementary material, which is available to authorized users.

## Background

*Shewanella* are well known for their extensively respiratory versatility and widely distributed in a range of aquatic habitats [1, 2]. Currently, the genus *Shewanella* is composed of more than 50 species [1], over 20 of which have abilities to reduce metal [3–5]. Due to their metal-reducing capability, some members of *Shewanella* such as *Shewanella oneidensis* MR-1, *Shewanella loihica* and *Shewanella putrefaciens* have been recognized as useful tools for bioremediation [6–8]. They were used to bio-remediate toxic elements and heavy metals contamination [9], and serve as biocatalyst in microbial fuel cells to produce H_2_ [10]. Despite their close evolutionary relationship, *Shewanella* strains from diverse habitats have great differences in genetic content [11]. *S*. *oneidensis* MR-1, a well-studied model of *Shewanella*, discovered to have the capability of heavy metal reduction in lakes, is the first species of *Shewanella* to be sequenced and assembled [12]. Some genes identified in *S*. *oneidensis* MR-1, such as *mtrBAC* and *omcA*, are essential for metal reduction [13, 14], but the features of their variations are still poorly understood. *S. putrefaciens* W3-18-1, a kind of psychrophile having the ability to reduce metals, has different molecular mechanisms of iron reduction from *S*. *oneidensis* MR-1 [15]. *S. piezotolerans* WP3 living at elevated hydrostatic pressures [16], also shows diverse electron transport pathways from *S*. *oneidensis* MR-1, although they have a close evolutionary relationship [17].

The pan-genome analysis provides a systematic way to assess the genomic diversity and evolution across diverse organisms [18, 19]. The availability of whole genome sequences for a number of *Shewanella* strains makes it possible to examine both the pan and core genome and provides insights into gene clusters of metal reduction in other members of this genus. A series of genome analyses of *Shewanella* strains have demonstrated gene diversity of electron acceptors for respiration [11, 20–22]. Investigations into several *Shewanella baltica* isolates have been conducted to analyze the complete genomic sequences and expressed transcriptomes [23], but only a few of the lineages are considered in genetic exchange analysis. Genome analysis of 10 closely related *Shewanella* strains suggested that variations in expressed proteomes correlated positively with the extent of environmental adaptation, but adaptive evolution in the core components of other species has still not been well studied [24]. Genome-wide molecular selection analyses, designed to assess selection pressure across the entire core-genome of different strains of *Shewanella* have not been reported, and also no comprehensive reports have attempted to address the important role of selection functions in the diversification of the core-genome of *Shewanella*. Most studies focus on identification of electron transport system and genes which are essential to the different metabolism [25]. To date, the whole genome sequence of certain *Shewanella* strains have been sequenced and some of their genetic features well characterized. However, only a limited number of strains have been extensively studied, the metabolic and genetic diversity of many species remain unknown. Therefore, a comparative pan-genome analysis in *Shewanella* is very necessary to study evolution and genetic diversity.

In this study, we estimated both the sizes of pan and core genomes, functional features, phylogeny, and horizontal gene transfer (HGT) to characterize population diversity and determine the forces driving adaptive evolution in 24 *Shewanella* strains. In addition, we attempted to assess selection pressure and selection functions in the diversification across the single copy core genomes within this genus. Furthermore, genetic organization and evolution of *mtr*–*omc* clusters were investigated for their evolutionary patterns at the genomic level.

## Results and discussion

### Core and pan-genome of *Shewanella*

**Fig. 1:**
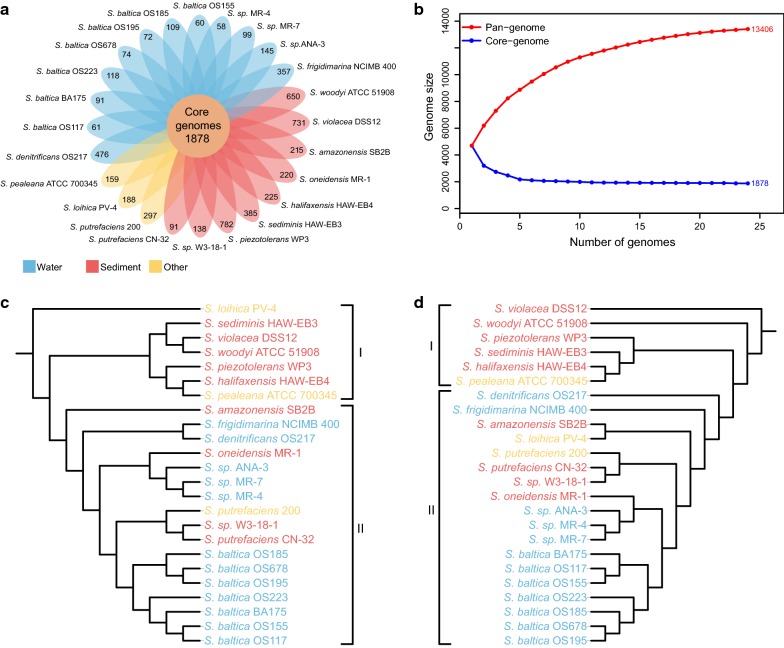
Genomic diversity of strains in *Shewanella*. **a** Core and specific families of 24 *Shewanella* genomes. Each oval represents a strain and is colored according to its isolation site (blue: water, red: sediment and yellow: other). The number of orthologous coding sequences (core genome) shared by all strains is in the center. The number of specific families is in non-overlapping portions of each oval. **b** Increase and decrease in gene families in the pan-genome (red) and core genome (blue), respectively. **c** Maximum likelihood phylogenetic tree based on 1788 single-copy gene families using 100 bootstrap replications. **d** Pan-genome tree based on presence/absence of gene family. The strain names are colored according to their isolation sites

A large proportion (94%) of genes from 24 *Shewanella* strains were grouped into 7830 homologous clusters (Additional file 1: Table S1). The pan-genome possessed 13,406 gene families, members of gene families in these strains were divided into three categories (core, accessory, specific) based on their appearance in different genomes. Among these gene families, 1878 families existed in all 24 genomes and hence represented the core gene complements (core genome). And 1788 families from core genome harbored only one gene from each strain (single-copy core families). Although these strains shared a core gene set, there were individual differences among the subsets of genes. The 5801 families existed in only one genome comprising the unique genes or singletons (strain-specific genome) and the remaining 5727 families (accessory genome) were present in more than one, but not all 24 genomes. The number of non-redundant strain-specific genes across different genomes varied from 58 to 782, and *S. piezotolerans* WP3 had the largest number of unique gene families (782) while *S.* sp. MR-4 possessed the smallest number of unique gene families (58) (Fig. 1a). This was consistent with the previous study that *S. piezotolerans* WP3 had the largest genome size among the sequenced *Shewanella* genomes [26]. The large proportion of specific genes suggested that the *Shewanella* strains harbored a high level of genomic diversity and uniqueness of each strain, showing their ability to survive in different environments. The size of pan-genome got large unboundedly with the increase of new genomes even including 13,406 non-redundant genes (Fig. 1b), which indicated that the *Shewanella* pan-genome was still “open”. This open pan-genome showed great potential for discovering novel genes with more *Shewanella* strains sequenced. In contrast to the pan-genome, the size of core genome appeared to reach a steady-state approximation after including 1878 non-redundant genes. In addition, only 14% of the pan-genome was found to be kept constant, while the remaining 86% was variable across the strains, which indicated that the pan-genome exhibited a high level of genome variability.

### Phylogeny of *Shewanella*

To gain insights into similarity and distance of the strains, two phylogenetic trees were constructed, one was based on the concatenated alignment of 1788 single-copy core genes (Fig. 1c), and another was based on absence or presence of each gene families (Fig. 1d). In the two trees, strains were grouped into two clades. The first clade was made up of most sediment strains, and the second one consisted mostly of water strains. *S. loihica* PV-4, isolated from iron-rich microbial mats [27], was phylogenetically distant from the rest strains, suggesting it had a low degree of similarity within core genes to the other strains. The separation tendency of different subgroups bore a high resemblance in the two trees in spite of the strains having different relative positions. *S. frigidimarina* NCIMB 400 showed much evolutionary relatedness to the *S. denitrificans* OS217 on the single-copy core gene tree, but they were no longer the sisters on the pan-genome tree, which indicated that non-core genes were likely to make them diverged. In addition, the *S. loihica* PV-4 was the sister with the *S. amazonensis* SB2B on the pan-genome tree, but had substantial distance on the single-copy core gene tree, suggesting that the variable genes made up a dominant proportion of the phylogenetic signal and played a role in the evolution of these two strains. It was also noted that those divergent strains, including *S. frigidimarina* NCIMB 400, *S. denitrificans* OS217, *S. amazonensis* SB2B, *S. violacea* DSS12, *S. woodyi* ATCC 51908, *S. sediminis* HAW-EB3, *S. halifaxensis* HAW-EB4, *S. pealeana* ATCC 700345, *S. piezotolerans* WP3 and *S. loihica* PV-4, contained more specific genes. Hence, it was likely that the larger differences of genes lead to evolutionary divergence, which suggested that different gene gain and loss might play important roles in the evolution of some *Shewanella* strains and make them apart from their relatives.

### CAZyme identification and profiling

**Fig. 2:**
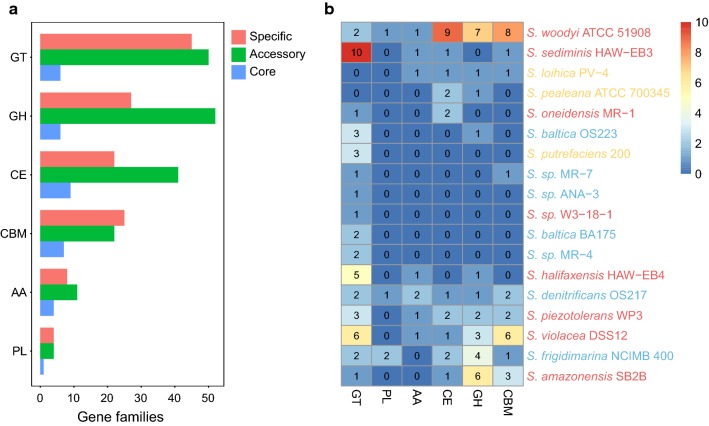
CAZyme distribution. **a** Distribution of CAZymes in pan-genome. The counts of orthologous genes assigned by CAZymes in the core genome (blue bars), the accessory genome (green bars) and the specific genome (red bars) are shown. **b** Distribution of specific genes assigned by CAZymes in each *Shewanella* strain. Strains are colored according to their isolation sites (blue: water, red: sediment and yellow: other). *GT* glycosyltransferases, *GH* glycoside hydrolases, *CE* carbohydrate esterases, *CBM* carbohydrate-binding molecules, *AA* auxiliary activities, *PL* polysaccharide lyases

Carbohydrate metabolism was one of the most important metabolic activities [28]. Members of *Shewanella* can anaerobically transfer electrons from cell metabolism to versatile electron acceptors [6]. The process of obtaining electrons from carbohydrates by *Shewanella* was the hydrolysis of complex carbohydrates present in biomass [29]. This was achieved through the presence of a repertoire of secreted or complexed carbohydrate active enzymes (CAZymes). To further understand the mechanism of polysaccharide hydrolysis in *Shewanella*, we identified the CAZymes in pan-genome. These strains contained an abundance of glycosyltransferases (GT), glycoside hydrolases (GH), carbohydrate esterases (CE), carbohydrate binding molecules (CBM), and a small number of auxiliary activities (AA) and polysaccharide lyases (PL) (Fig. 2a). In addition, these CAZymes were more abundant in accessory and specific genome than in core genome. This diversification of the *Shewanella* CAZyme-encoding genes allowed for different electron-transporting roles in cellular metabolism. In addition, strains varied in their number of unique CAZyme-encoding genes, suggesting the metabolic abilities of carbohydrates were specialized among *Shewanella* strains. There were 18 strains possessing specific genes for CAZymes, and GTs and GHs were most abundant (Fig. 2b).

Additionally, we found that strains isolated from the water seemed to contain less specific CAZyme-encoding genes than other strains from more diverse environments. The diversity of such specific CAZyme-encoding genes was important to the strategies of carbohydrate metabolism that would result in metabolism specialization and environment adaptation. The *S. woodyi* ATCC 51908 had the most strain-specific CAZymes (28), followed by *S. violacea* DSS12 (17) and *S. sediminis* HAW-EB3 (13). The three strains had more strain-specific CAZyme-encoding genes and they were isolated from detritus or sediment. Such high number of strain-specific CAZyme-encoding genes might contribute to their metabolic diversity in more diverse environments. Furthermore, GTs were the most common in the unique genes, such patterns suggested that GTs might be important in the environmental adaptation of these strains. However, GEs, CBMs and GHs were the most abundant among the *S. woodyi* ATCC 51908 unique genes, suggesting that GEs, CBMs and GHs might be important in the detritus adaptation of *S. woodyi* ATCC 51908.

### Functional profiling of pan-genome

**Fig. 3:**
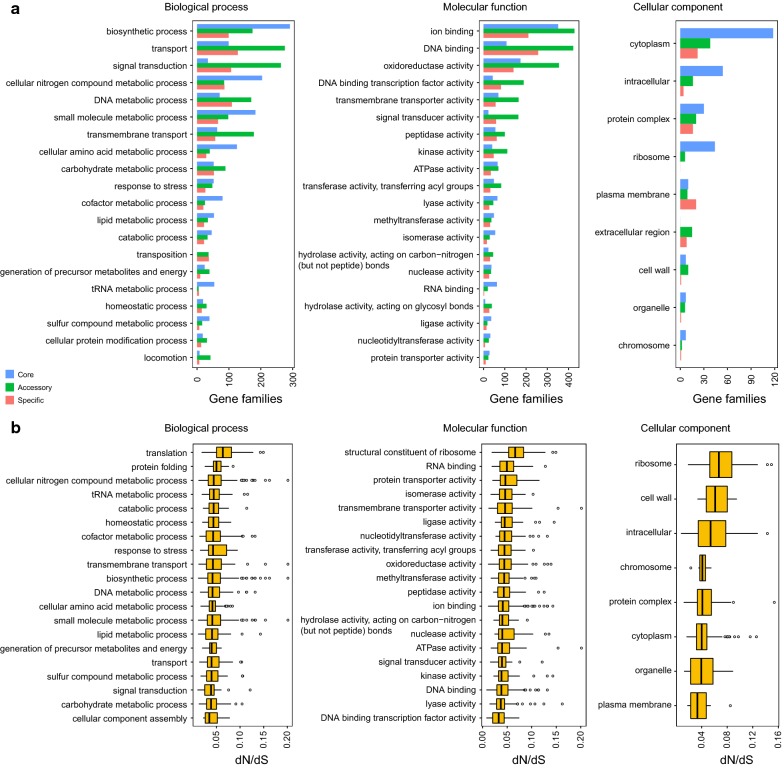
GO annotation of gene families. **a** GO annotation of pan-genome in biological processes, molecular function and cellular component. Core clusters (blue), accessory clusters (green) and specific genes (red). **b** Distribution of selection pressures by categories of biological processes, molecular function and cellular component. Values of dN/dS assigned by GO categories in the single-copy core genome are showed

To gain insights into functional diversity in these strains, the pan-genome from the 24 genomes was also compared using functional gene ontology categories. All of the GO terms fell into three categories: biological processes (BP), molecular function (MF) and cellular component (CC) (Fig. 3a). Genes with biosynthetic process, transport and signal transduction were more abundant in the BP category. Genes related to ion binding, DNA binding and oxidoreductase activity were enriched in MF category. For the CC category, genes involved in cytoplasm, intracellular and protein complex were dominant. Pan-genome processed a great number of genes involved in various metabolic pathways and enzymes. The presence of so many carbon and energy utilization pathways, material transport and synthesis systems was a reflection of the high-pressure and deep-sea extreme environment. In addition, the existence of so many genes involved in transport, transmembrane transport and ion binding provided supports for iron reduction. The vast majority of genes in the core genome were involved in housekeeping functions, such as biosynthetic process, metabolic functions, cytoplasm, intracellular and ribosome (Fig. 3a). These categories were also present, but hardly appeared in the accessory and specific genome, whereas the accessory and specific genes were enriched in transport, signal transduction, binding and enzymatic activity (Fig. 3a). The non-core genes could be important for adaptation to specific habitats. Such functional enrichment patterns of accessory and specific genes reflected their notable respiratory diversity.

In addition, cluster of orthologous group (COG) functions of the pan-genome were compared. Genes with predicted functions and unknown functions were more abundant in the pan-genome (Additional file 2: Figure S1). The majority of genes in the core genome were involved in ‘Energy production and conversion’ (C), ‘Amino acid transport and metabolism’ (E) and ‘Translation, ribosomal structure and biogenesis’ (J) (Additional file 2: Figure S1). Whereas the accessory and specific genome had a slight increase in genes involved in ‘Amino acid transport and metabolism’ (E), ‘Transcription’ (K) and ‘Signal transduction mechanisms’ (T) (Additional file 2: Figure S1).

To reveal the uniqueness of the strains, we detected several specific genes that might be related to the survival environment. Among the strain-specific genes in pan-genome, *S. loihica* PV-4 had a gene encoding menaquinone biosynthesis protein MenD that facilitated electron transfer. Previous study suggested that menaquinone was the electron transporter in the respiratory chain and was essential for the survival of *S. loihica* PV-4 [27]. This *menD* gene did not exist in other strains, which might be important for the survival of *S. loihica* PV-4 in the iron-rich environment. In addition, *S. woodyi* ATCC 51908, *S. pealeana* ATCC 700345, *S. sediminis* HAW-EB3 and *S. halifaxensis* HAW-EB4 possessed one or two strain-specific genes encoding nitrite reductase that were involved in nitrogen oxide reduction. The *S. amazonensis* SB2B, also had a specific gene encoding glutamine synthetase that was a key enzyme of nitrogen metabolism. It was reported that *Shewanella* was capable of nitrate reduction using nitrate as the electron acceptor under certain conditions [30]. These five strains had such strain-specific genes related to nitrogen utilization, which indicated that they had characteristics that were more complex in response to nitrogen. In addition, *S. amazonensis* SB2B and *S. sediminis* HAW-EB3 possessed genes encoding inorganic diphosphatase. The specificity of this enzyme has been reported to vary with the source and the activating metal ion [31]. *S. violacea* DSS12 had a specific gene-encoding related cytochrome oxidase that was responsible for oxidative phosphorylation. This gene was adjacent to other electron transfer genes in *S. violacea* DSS12 genome, suggesting that it might interact with those adjacent genes and function in the related pathways.

### Selective pressure analysis

To investigate conservation and evolution of housekeeping genes, functional diversification and evolutionary pressure of single-copy core genes were detected. In addition, the non-synonymous (dN) to synonymous (dS) substitution rates (dN/dS) were estimated for each single-copy core family. The analysis showed that all of the single-copy core genes encountered a strong purifying selection (Fig. 3b). Such an important pattern of strong purifying selection indicated that the single-copy core genes were highly conservative and purifying selection contributing to their long-term stability.

The combination of selective pressure and the functional categories could reveal the functions involved in rapid evolution. We estimated the strength of purifying selection for single-copy genes in different functions. Genes tended to exhibit different degrees of conservative evolutionary directions, and some of them experienced weaker purifying selection especially those involved in translation, protein folding and structural constituent of ribosome (Fig. 3b). However, genes involved in biological processes of cellular component assembly evolved under strongest purifying selection. For different categories of molecular function, genes undergoing the strongest purifying selection were those involved in DNA binding transcription factor activity, while genes involved in structural constituent of ribosome underwent more relaxed purifying pressure. Furthermore, genes involved in ribosome exhibited higher evolutionary rates, while genes involved in plasma membrane exhibited lower evolutionary rates. These results suggested that genes undergoing higher purifying selection, played dominant roles in the evolutionary rates among the *Shewanella* strains, making them retain the original functional process of cellular component assembly, plasma membrane, etc. This observation of purifying selection in single-copy core genes suggested that purifying selection might drive evolution in the essential life functions across all 24 *Shewanella* strains.

### Gene gain and loss

**Fig. 4:**
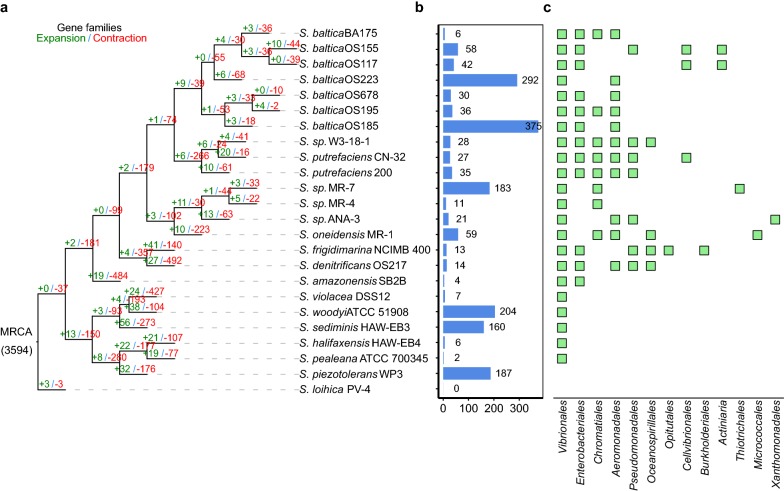
Dynamic evolution of orthologous gene family. **a** Gene family expansion and contraction in each evolutionary branch. Phylogenetic tree is constructed by 1788 single-copy gene families. The number of expanded (green) or contracted (red) gene families in each strain is on the corresponding branch. MRCA: most recent common ancestor. **b** Distribution of recent horizontal genes in each strain. **c** The presence of HGT genes in donor bacteria

Gene gain and loss during evolution can increase the fitness of bacteria within habitats [32, 33]. To obtain deeper insight into the evolution of gene families over a phylogeny, we performed an analysis of gene expansion and contraction at each branch of speciation. Diversification at branches was associated with a large number of changes in gene family size across the *Shewanella* tree. The 3594 gene families were more likely to have been inherited from the most recent common ancestor (MRCA) of *Shewanella*, and 1805 of which (50.22%) families experienced size changes (Fig. 4a). In the tree, each strain branch of the phylogeny has changed in evolution (Fig. 4a). In addition, *S. loihica* PV-4, the most distantly related strain based on single-copy phylogeny, had only six changed genes. Furthermore, contractions outnumbered expansions on all branches except the branch of *S. loihica* PV-4, *S. putrefaciens* CN-32, and *S. baltica* OS195. An average of 15 gene families was under expansion, whereas 123 gene families contracted in each strain branch, indicating that loss function played an important role in dynamic evolution. The gene families expanded in those branches of strains were mainly enriched in categories of oxidation–reduction process, catalytic activity and membrane. (Additional file 3: Figure S2A). Gene enrichment analysis showed that these strains have lost the majority of genes involved in oxidation–reduction process, DNA binding and membrane (Additional file 3: Figure S2B). The enrichment of changed genes in reduction process and membrane seemed to be ecologically important and contribute to the successful adaptation of the strains.

Due to recent acquisition and deletion, the gene that is not in the MRCA is more variable than the core gene [34]. HGT is the main driver of bacterial evolution and adaptation [35]. To infer the recent evolutionary dynamics of *Shewanella* on a larger scope of strains, we examined all horizontally acquired genes and obtained 1800 genes that were of horizontal origin and the number of HGT-origin genes detected each strain was different (Fig. 4b). Gene transfer occurred in many genes, which indicated that horizontal transfer genes played an important role in the gene flow and acquisition and contributed to the open pan-genome of the *Shewanella*. Among these strains, *S. baltica OS185* appeared to gain the largest number of genes, whereas *S. loihica PV*-*4* did not detect the horizontal gene. *S. loihica PV*-*4* showed closest evolutionary relatedness in core gene similarity to the nearest common ancestor, and the numbers of gene expansion, contraction and HGT genes were minimum, probably because the strain’s living environment is similar to the ancestor. To infer the contribution of donors to horizontal gene transfer, we identified donors for these horizontal transfer genes (Fig. 4c, Additional file 4: Table S2). These transferred genes appeared to originate from members of *Vibrionales* and *Enterobacteriales* family, particularly the genera *Vibrio*, which suggested that HGT events were more effective to occur in closely related organisms rather than in distantly related organisms. In addition, these HGT-origin genes were enriched in DNA metabolic process, transposition and DNA binding (Additional file 3: Figure S2C). The acquisition of genes in DNA metabolic process, transposition and DNA binding could aid in their survival in different environments. For example, the *S. woodyi* ATCC 51908, *S. sediminis* HAW-EB3, *S.* sp. MR-4 and *S.* sp. MR-7 acquired two or three genes encoding the nitrate reductase. The *S. woodyi* ATCC 51908, *S. sediminis* HAW-EB3, *S. baltica* OS185, *S. baltica* OS223 and *S. piezotolerans* WP3 strains acquired a gene encoding glutamine synthetase. These genes were involved in the nitrogen cycle, which was apparently important for the successful adaptation. These results suggested that HGT was an important driver of the evolution of these genomes. Therefore, the available gene pool for HGT could explain the difference in non-MRCA genomes and the increased pan-genome size of these strains. These findings suggested that gain and loss of genes that were apparently accessory for the majority of the strains of a genus might be a successful strategy for biological diversification, rapid evolution and environmental adaptation.

### Evolution of the *mtr* clusters in *Shewanella*

**Fig. 5:**
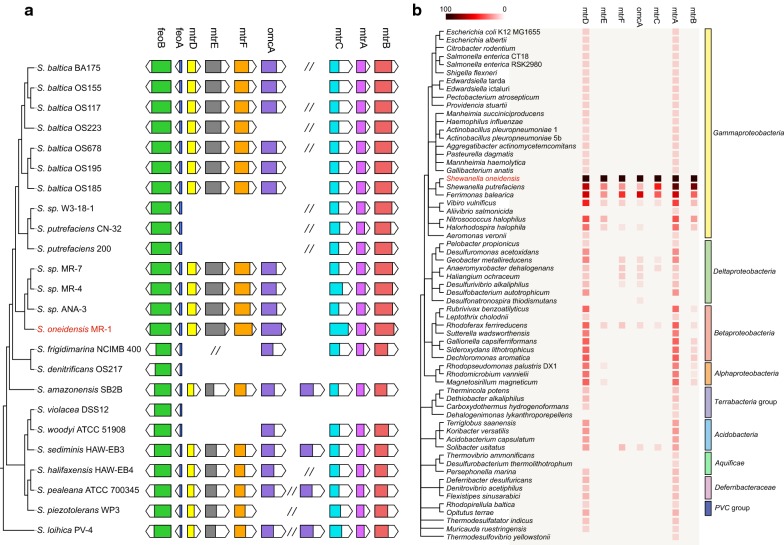
Global comparison of the *mtr*–*omc* clusters in *Shewanella* and orthologous gene clusters in other strains. **a** Genetic comparative organization of the gene locus associated with the metal-reducing pathway of 24 *Shewanella* strains. Arrows indicate genes and their orientations. The length of the colored box indicates gene homology with those in MR-1. The double slash indicates the existence of extra genes. **b** The genetic occurrence of the *mtr* homologous clusters in diverse bacteria. The tree on the left is a taxonomic common tree, and the bars on the right represent the classifications of bacteria. *S. oneidensis* MR-1 is colored in red

Previous studies on *S. oneidensis* MR-1 indicated that genes within a cluster (*mtrBAC*–*omcA*–*mtrFED*) were involved in the metal-reducing pathway, while the *S. denitrificans* OS217 did not possess this cluster [17, 36]. In particular, this cluster comprised seven genes that were clustered in sequential order of *mtrD*–*mtrE*–*mtrF*–*omcA*–*mtrC*–*mtrA*–*mtrB* [37]. The *feoA*–*feoB* operon involved in ferrous iron transport was common to all strains and adjacent to the *mtr*–*omc* cluster. To explore new features of metal reduction pathways, we compared this kind of *mtr*–*omc* gene cluster in these 24 strains. Although genes within this cluster shared a high degree of similarity with the homologues described in *S. oneidensis* MR-1, the number and composition carried out by different *Shewanella* strains were still changed (Fig. 5a). The complete *mtrBAC*–*omcA*–*mtrFED* cluster was conserved in 15 out of the 24 strains. In addition, the *mtrABC* operon was conserved in all the genomes except for *S. denitrificans* OS217 and *S. violacea* DSS12. The *mtrDEF* operon, homologues of *mtrABC*, was virtually absent from *S.* sp. W3-18-1, *S. putrefaciens* CN-32, *S. putrefaciens* 200, *S. frigidimarina* NCIMB 400, *S. denitrificans* OS217, *S. violacea* DSS12 and *S. woodyi* ATCC 51908. The *mtr*–*omc* clusters of 17 strains were considerably different from that of *S. oneidensis* MR-1 since there were gene duplication, gene loss, or new gene gains within it. Such change of this cluster reflected the evolutionary history and possibly metal respiratory specialization, which agreed well with the previous study that the absence of genes in this *mtr*–*omc* cluster would result in slower iron reduction [38]. For example, *S. denitrificans* OS217 and *S. violacea* DSS12, which both lacked the entire cluster, showed limited anaerobic growth capacity that may be due to gene loss in the process of ecological specialization [4]. In addition, expansion of *omcA* gene occurred in *S. amazonensis* SB2B, *S. sediminis* HAW-EB3, *S. pealeana* ATCC 700345 and *S. loihica PV*-*4*, which reflected gene acquisition and dynamic evolution in metal respiration. The tree of genes within the cluster and the *cymA* gene showed that each gene formed a group with its orthologs from different strains (Additional file 5: Figure S3). Branches of each gene clustered into two distinct groups, which imply two distinct evolutionary paths for different strains. In addition, the evolutionary relationship of *omcA* between these strains was determined according to their genetic characteristics. These *S. baltica* strains showed much evolutionary relatedness in each gene, and the *omcA* genes from multi-locus in *S. loihica* PV-4, *S. sediminis* HAW-EB3 and *S. pealeana* ATCC 700345 formed the relatively independent branch, showing the duplication events (Additional file 6: Figure S4).

The presence of *mtr*–*omc* cluster in *Shewanella* was supposed to correlate with its deep-sea habitat, this cluster presumably shared with other deep-sea microorganisms [39]. To examine extensive genetic exchange between dissimilatory metal-reducing bacteria, the genetic occurrence of the *mtr* homologues was predicted (Fig. 5b). The apparent widespread distribution of metal-reducing pathways in other bacteria indicated the importance of electron-transfer pathway in microbial oxidation–reduction of iron [37]. The cluster facilitating the iron respiration was conserved among closely related species such as *Ferrimonas balearica* and *Vibrio vulnificus*, and showed the same sequential order of *mtrC*–*mtrA*–*mtrB*, indicating that the order of the homologues genes was highly conserved. This was in accordance with the previous study that homologues of the metal-reducing pathway were found in a series of other dissimilatory metal-reducing bacteria and might have co-evolved in these bacteria [40]. The protein products of these *mtr* genes worked together to facilitate electron transfer across the cell envelop [37], which had been recognized as an early form of respiration, although it was widespread among the bacteria [41], there were still fewer strains containing complete orthologous genes of metal reduction system. The genes *mtrA* and *mtrD* were present in most strains, but few *mtrBCEF* and *omcA* genes were present. The lack of apparent *mtr* genes in the most metal-reducing strains was consistent with previous studies that the electron-transfer pathways used by metal-reducing strains for extracellular reduction of Fe(III)-containing minerals had evolved independently [17].

**Fig. 6:**
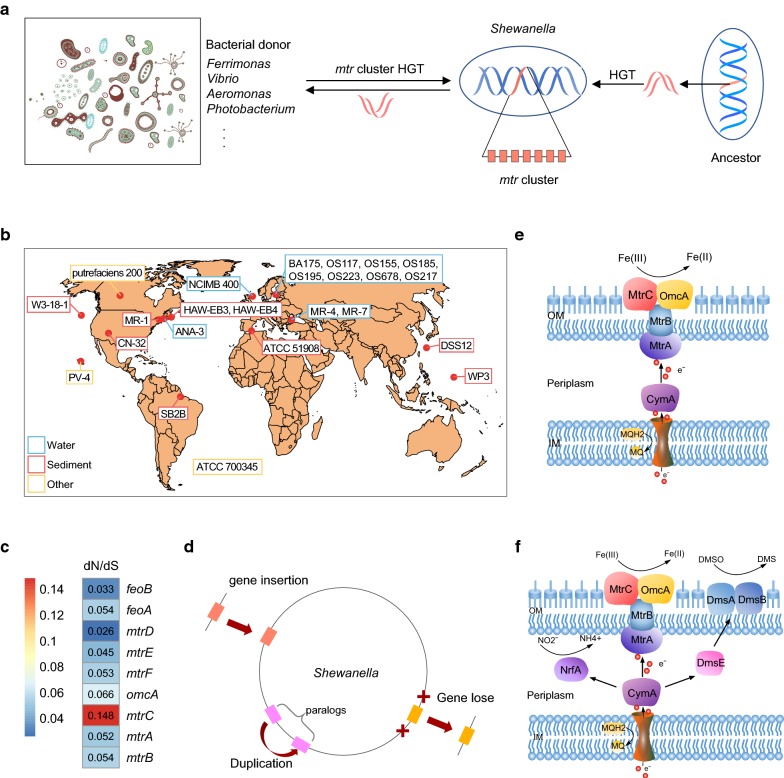
A hypothetical model for the evolution of the Mtr pathway in *Shewanella*. **a** *Shewanella* may acquire the *mtr* genes from the ancestor by HGT and exchange with other bacteria. **b** The geographic origin of 24 *Shewanella* genomes used in this study. **c** Selection pressure of *mtr* genes, with each box showing the ratio of nonsynonymous (dN) over synonymous substitution (dS) rate. Red boxes represent genes that with high ratios, blue boxes represent genes that with low ratios. **d** Dynamic changed of *mtr* clusters. Gene gains and losses derived from HGT events and genes duplication. **e** The metal-reducing pathway of *S. oneidensis* MR-1 (*OM* outer membrane, *IM* inner membrane, *MQH2* reduced form of menaquinone, *MQ* oxidized form of menaquinone). **f** The depiction of multiple electron transfer pathways of respiration reported in *Shewanella*

The metal-reducing pathway was generally considered as one of the most ancient microbial metabolisms on earth a long time ago [39]. Previous study had described that *Shewanella* contained regions, which were likely to be horizontally acquired from members of the Enterobacteriaceae family bacteria [4]. To infer the evolutionary relationships of *mtr*–*omc* cluster on a larger scope of strains, individual phylogenetic trees of *mtr* genes from different organisms were constructed (Additional file 7: Figure S5). Phylogenetic trees of *mtrE*, *mtrF*, *mtrA* and *mtrD* showed that *Shewanella* strains exhibited very similar evolutionary relatedness among each other and were relatively independent from other bacteria. However, several *Shewanella* strains were scattered to different branches in phylogenetic trees of *mtrB*, *mtrC*, *omcA* (Additional file 7: Figure S5). These *mtr* genes of *Shewanella* strains showed evolutionary relatedness to *Ferrimonas* species, probably because of the extensive horizontal gene transfer events (Fig. 6a). In addition, the differences of the metal-reducing pathway of *Shewanella* strains might be due to the result of multiple gene gains and loss events during the evolution. As these isolates originated from diverse geographic locations and habitats, such as marine water, sediments and subsurface, they carried out a diverse range of metabolic processes (Fig. 6b). And the *mtr* genes had changed during the evolution since they were under strong purifying selection (Fig. 6c). The strong purifying selection played a key role in selecting for such iron respiration changes in their evolution. The *mtrC* had lower similarity with the homologues compared with other genes, underwent more diversifying selection. Besides, due to the constraint of purifying selection, some of the *mtr* experienced gene loss and gene duplication, as well as new gene gains (Fig. 6d). In the metal-reducing pathway, electrons from the inner membrane passed through the periplasm and across the outer membrane to the extracellular minerals via the protein components encoding by *mtr*–*omc* cluster [42] (Fig. 6e). With the gain and loss of genes, genes of *mtr*–*omc* cluster were no longer core genes, and the ability for *Shewanella* to adapt to environmental conditions by metal-reducing pathway was presumably no longer essential for survival. Another possible explanation could be that other pathways had been developed to utilize different types of electron acceptors during their evolution to facultative anaerobic environments [43] (Fig. 6f). In particular, *mtr* genes experienced additional contraction events with some of them lost during evolution, which indicated that respiration had become less competitive as the environment changed.

## Conclusions

In this study, a comparative pan-genome analysis has been conducted to study the genomic diversity and evolutionary relationships among these 24 *Shewanella* strains. The pan-genome exhibited a high level of genome variability, about 86% of which was variable. And this pan-genome was found to be open and had a high percentage of strain-specific genes within it. Moreover, the existence of several strain-specific genes and abundant CAZymes in the accessory and specific genomes reflected the functional diversity and metabolic diversity of the pan-genome. And also the essential function of single-copy core genes would evolve under purifying selection, which illustrated the importance of the housekeeping function to each strain for survival. We also found that HGT played a key role in the shaping of the *Shewanella* accessory and specific genome and accelerated the evolution of strains. Furthermore, purifying selection played an important role in the stability of the core-genome and facilitated the evolution of *mtr*–*omc* clusters in different *Shewanella* strains to different habitats. We re-emphasized that the number of *Shewanella* strains was expanded to 24, which had not been reported by others in the previous studies.

The work presented here would help carry out further research on the genetic basis of *Shewanella*, and better enhance understanding of metal-reducing pathway and the impact of HGT on the evolution. It could also contribute to research on applications of metal-reducing bacteria in bioremediation and metabolic engineering.

## Methods

### Datasets

The genomic features, geographical origin and isolation site characteristics of the genomic sequences used in this study are provided in Additional file 8: Table S3. Genomes and protein sequences were downloaded from National Center for Biotechnology Information (NCBI), representing 24 different strains.

### Gene family

To understand the evolutionary relationship of *Shewanella*, we conducted systematic comparative genomic studies. Full protein-coding genes of 24 *Shewanella* strains were used to construct gene families using OrthoMCL (v2) [44] with a BLAST *E*-value cut-off of 1*e*−5 and an inflation parameter of 1.5. Here, the clustering results yielded 7830 homologous clusters, 1788 of which were single-copy gene families, and then they were parsed and concatenated.

### Phylogeny construction

Protein sequences for these single-copy gene families were concatenated and aligned by MUSCLE(v3.8) [45] with default parameters. The alignments were curated by GBlock (v0.91b) [46] to filter out poorly aligned positions. The phylogenetic tree was constructed using the maximum likelihood algorithm with 100 bootstraps as implemented in Phylip (v3.696).

The pan-genome tree based on the absence or presence of each gene family in all genomes used the Manhattan distance to measure the evolutionary relationship of strains. Each gene in the genome was scored on basis of the presence (1 value) or absence (0 value), a 0/1 matrix was built and Manhattan distance was calculated, then a phylogenetic tree of the pan-genome was generated using MEGA (v5).

To determine the evolutionary origin of *mtr*–*omc* clusters, the co-occurrence of *mtr*–*omc* clusters in bacteria was obtained from the STRING [47] database. In addition, homologous *mtr* genes identification from other genus were based on the best match of the alignment to the NCBI non-random protein database using the BLASTp program with an *E*-value cut-off of 1*e*−5. Then the Phylip program was used to infer the phylogenetic relationships among these mtr genes.

### Gene distribution expansion

To gain a great insight into the evolutionary dynamics of the genes, the expansion and contraction of the gene families among these 24 *Shewanella* strains were determined. CAFE (v2.1) [48] was used to infer the change in gene family size in each branch. Gene gain and loss were along with each branch of the single-copy gene family tree, and significant levels of expansion and contraction were determined at 0.05.

### Detection of horizontal gene transfer

To infer mobile elements of *Shewanella* genome, all non-recent common ancestral genes were aligned to plasmid sequences, insertion sequences, phage sequences available in the NCBI RefSeq database, ISfinder and ACLAME database, respectively. A gene was used as input for HGTector (v0.2.1) [49] searches in terms of its best BLASTp hit with a sequence identity of above 50% and a cutoff *E* value of 10^−5^. The identification of HGT-origin genes used HGTector with BLAST parameter thresholds 90% identity, an *E*-value of 10^−5^ and 500 top-scoring matches. The *Shewanella* and *Shewanellaceae* were set as self-group and close group, respectively.

### Functional annotations

Hmmscan was used to determine carbohydrate activity enzymes (CAZymes) by comparing all gene families to dbCAN [50] database. Gene ontology (GO) terms were identified using InterProScan (v.5) [51]. GO term assignments for each of the genes were retrieved from InterproScan results. Since GO slims were particularly useful for giving a summary of the genome-wide GO annotation, all of the GO terms were mapped to GO slim (http://www.geneontology.org/GO.slims.shtml). Additionally, gene annotation was based on COG databases, using BLAST with a cutoff *E*-value of 1*e*−5.

### Selective pressure analysis

To estimate the rate of evolution and test the selection pressure on each single-copy orthologous genes, the program PAML (v. 4.4c) [52] was used. For each pair of strains, the Codeml model was used to calculate dN and dS values. The single *ω* (dN/dS, the ratio of non-synonymous to synonymous divergence) across sites was estimated using M0, M7, M8 models that were fixed across the phylogeny for each alignment (referred to as *ω* of a gene). To avoid convergence problems, each analysis was repeated three times with different initial values of *ω* and adopted results from the analysis with the highest likelihood.

## Additional files

**Additional file 1: Table S1.** Summary of homologous genes identified by OrthoMCL. Protein sequences from 24 *Shewanella* genomes were used as input for OrthoMCL orthologous gene clustering (Input Genes). Clusters containing single genes or genes from a single strain were eliminated (Clusters). Genes from a single strain (Clustered as single strain).

**Additional file 2: Figure S1.** Distribution of genes based on COG category of the pan-genome. Distribution of COG categories between the core (blue bars), accessory (green bars) and specific genes (red bars) of *Shewanella.*

**Additional file 3: Figure S2.** GO annotation of gained and lost genes. (A) Function enrichment of expanded gene families. (B) Function enrichment of contracted gene families. (C) Function enrichment of HGT-origin genes.

**Additional file 4: Table S2.** Possible donors of HGT genes in each strain.

**Additional file 5: Figure S3.** Phylogenetic tree of *mtr*–*omc* gene clusters in 24 *Shewanella* genomes. The phylogenetic tree was constructed using a multiple sequence alignment of the full *mtr*–*omc* clusters and *cymA* genes of all 24 *Shewanella.*

**Additional file 6: Figure S4.** Comparison of *omcA* genes in 24 *Shewanella* genomes. Left: Phylogenetic tree of *omcA* genes. Right: *omcA* genes similarities of pairwise comparison. The *omcA* from multi-locus that are not located in the *mtr*–*omc* cluster are colored red, *omcA* from multi-locus that located in the *mtr*–*omc* cluster that no longer sisters are colored green, respectively.

**Additional file 7: Figure S5.** Phylogenetic relationship of *mtr*–*omc* clusters within different bacteria. The phylogenic trees built by protein sequences were showed for (A) *mtrB*, (B) *mtrC*, (C) *mtrE*, (D) *mtrF*, (E) *omcA*, (F) *mtrA* and (G) *mtrD*, respectively. The branches of *Shewanella* and clade of *S. oneidensis* MR-1 are marked in red.

**Additional file 8: Table S3.** Genomes and strains used in this study. Organism information is collected from NCBI.
