# Supplementary figures and images for: Pan-genome analyses of 24 Shewanella strains re-emphasize the diversification of their functions yet evolutionary dynamics of metal-reducing pathway
Source: Biotechnol Biofuels. 2018 Jul 17;11:193. doi: 10.1186/s13068-018-1201-1 (PMC6048853; doi:10.1186/s13068-018-1201-1)

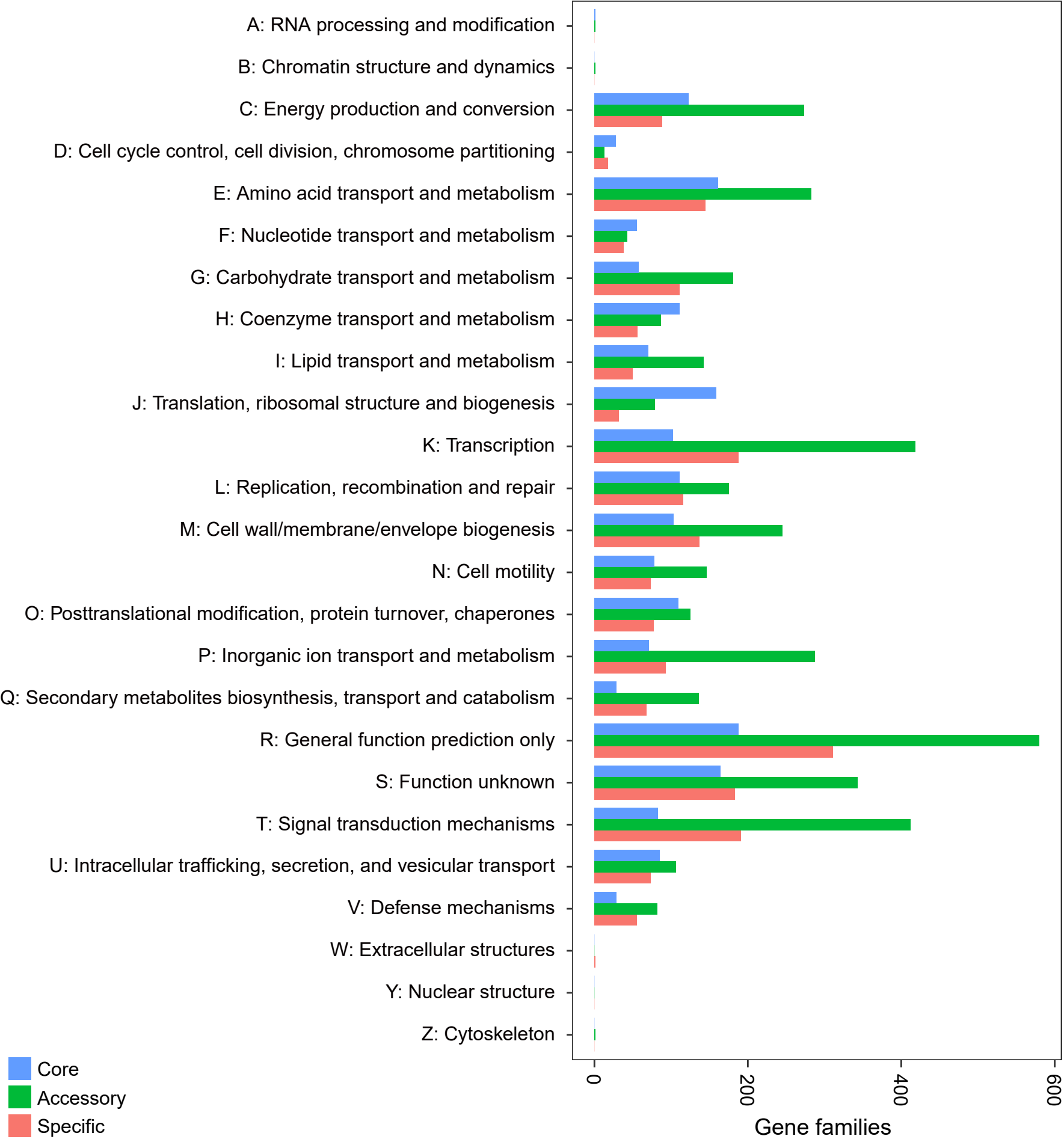

Supplement: Supplementary file 2 — Additional file 2: Figure S1. Distribution of genes based on COG category of the pan-genome. Distribution of COG categories between the core (blue bars), accessory (green bars) and specific genes (red bars) of Shewanella. [file 13068_2018_1201_MOESM2_ESM.pdf]

(A)

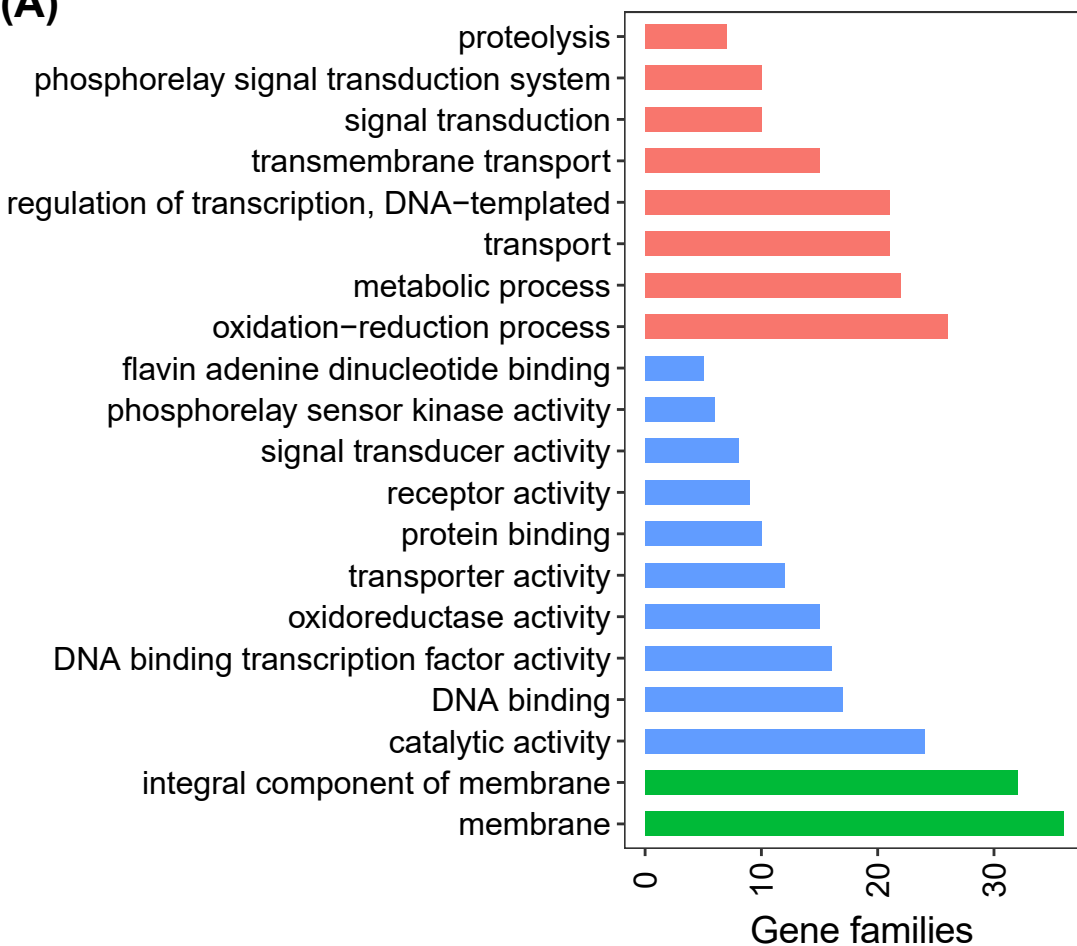

(B)

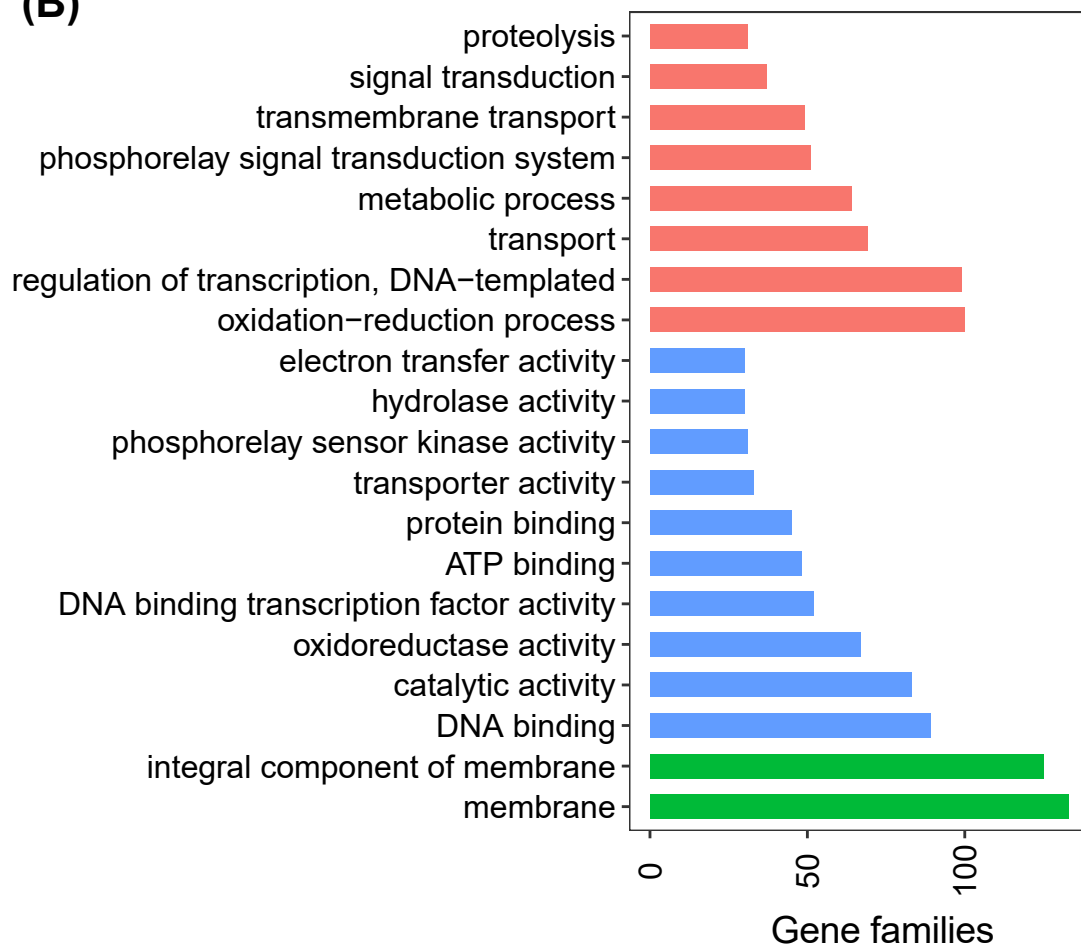

(C)

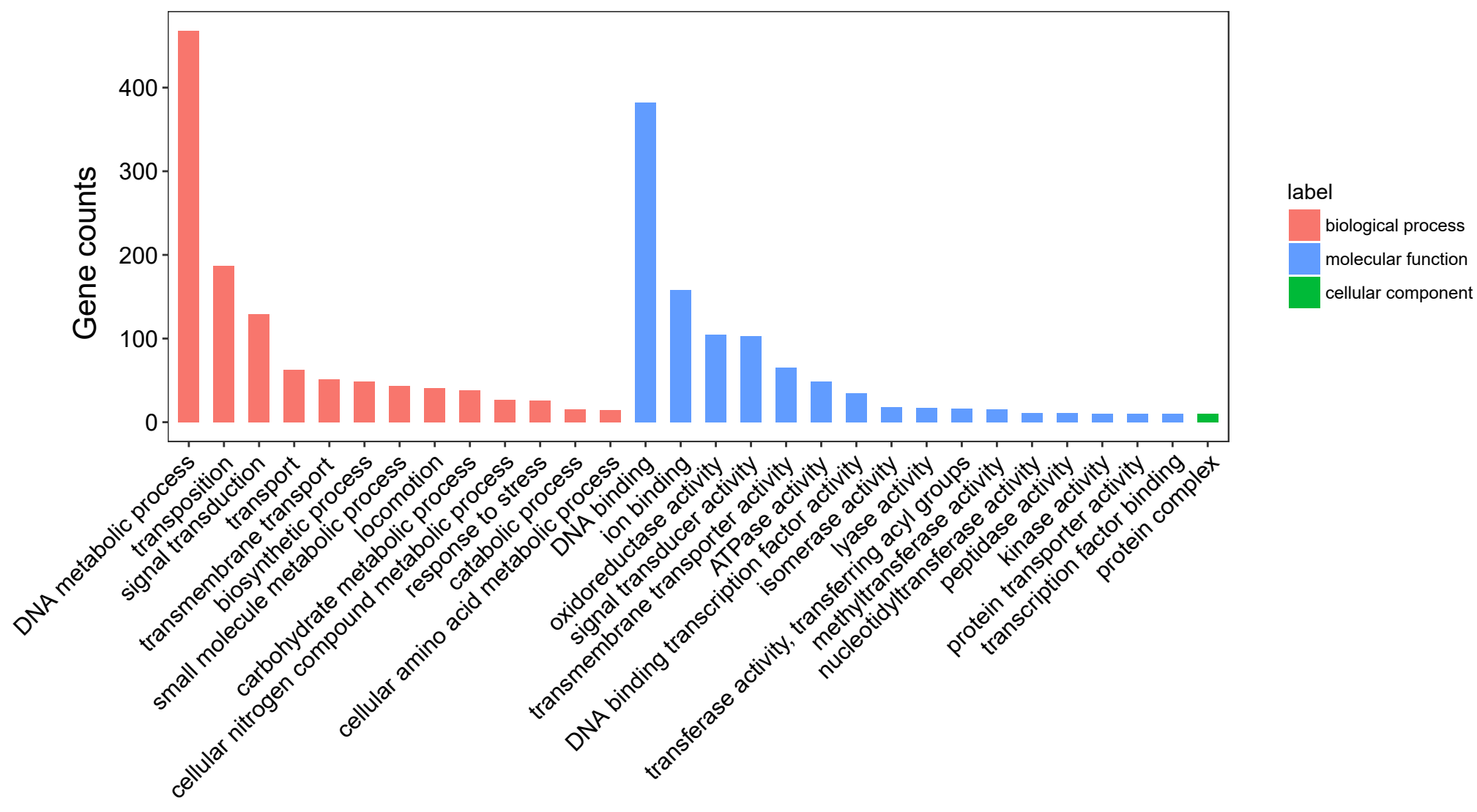

Supplement: Supplementary file 3 — Additional file 3: Figure S2. GO annotation of gained and lost genes. (A) Function enrichment of expanded gene families. (B) Function enrichment of contracted gene families. (C) Function enrichment of HGT-origin genes. [file 13068_2018_1201_MOESM3_ESM.pdf]

|                                                                                 |      |
|---------------------------------------------------------------------------------|------|
| 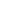 | mtrA |
| 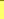 | mtrD |
| 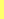 | cymA |
| 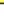 | mtrC |
| 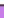 | mtrF |
| 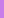 | omcA |
| 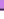 | mtrE |
| 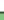 | mtrB |

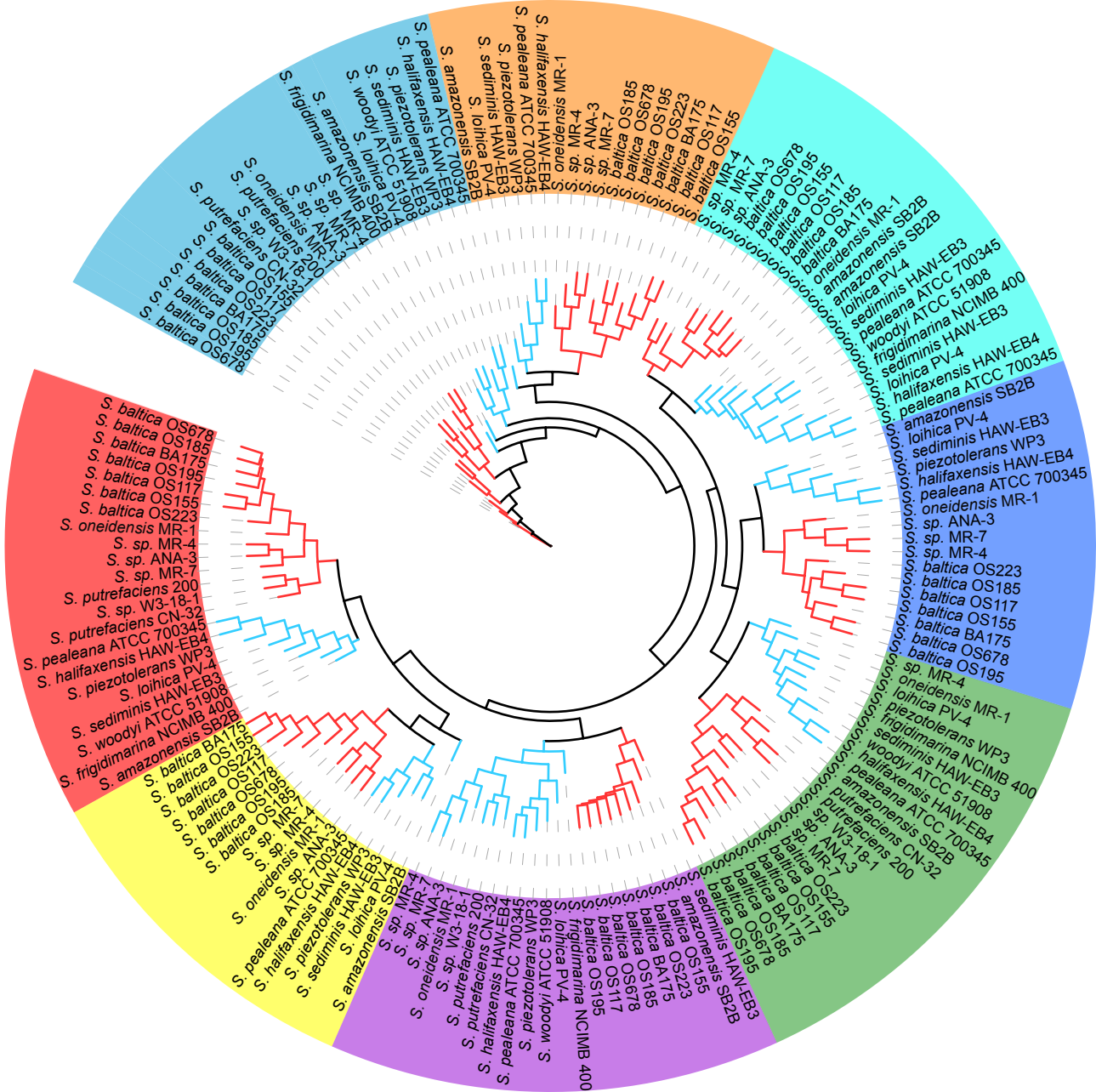

Supplement: Supplementary file 5 — Additional file 5: Figure S3. Phylogenetic tree of mtr–omc gene clusters in 24 Shewanella genomes. The phylogenetic tree was constructed using a multiple sequence alignment of the full mtr–omc clusters and cymA genes of all 24 Shewanella. [file 13068_2018_1201_MOESM5_ESM.pdf]

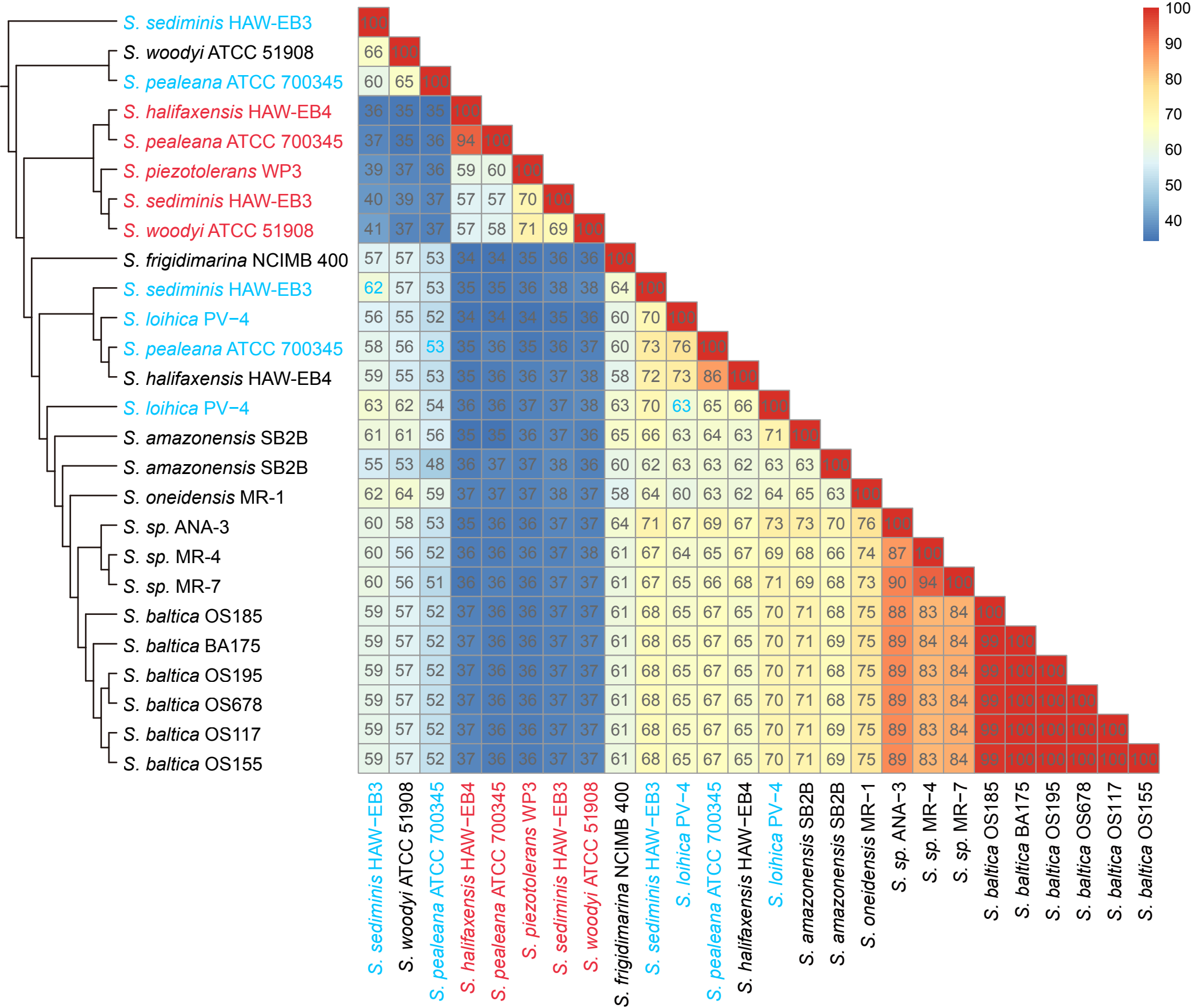

Supplement: Supplementary file 6 — Additional file 6: Figure S4. Comparison of omcA genes in 24 Shewanella genomes. Left: Phylogenetic tree of omcA genes. Right: omcA genes similarities of pairwise comparison. The omcA from multi-locus that are not located in the mtr–omc cluster are colored red, omcA from multi-locus that located in the mtr–omc cluster that no longer sisters are colored green, respectively. [file 13068_2018_1201_MOESM6_ESM.pdf]

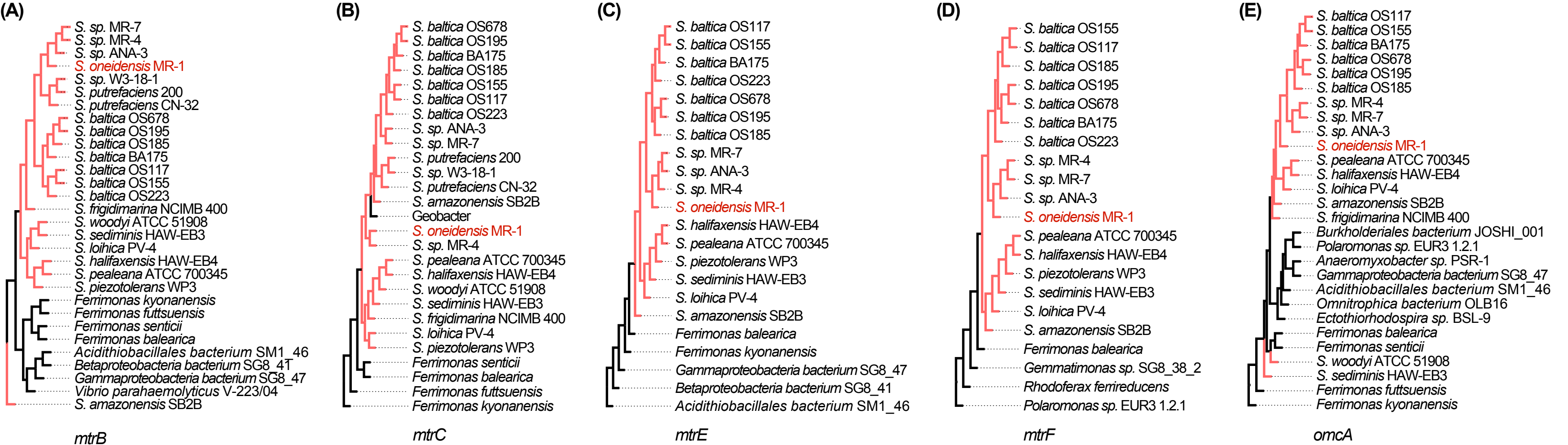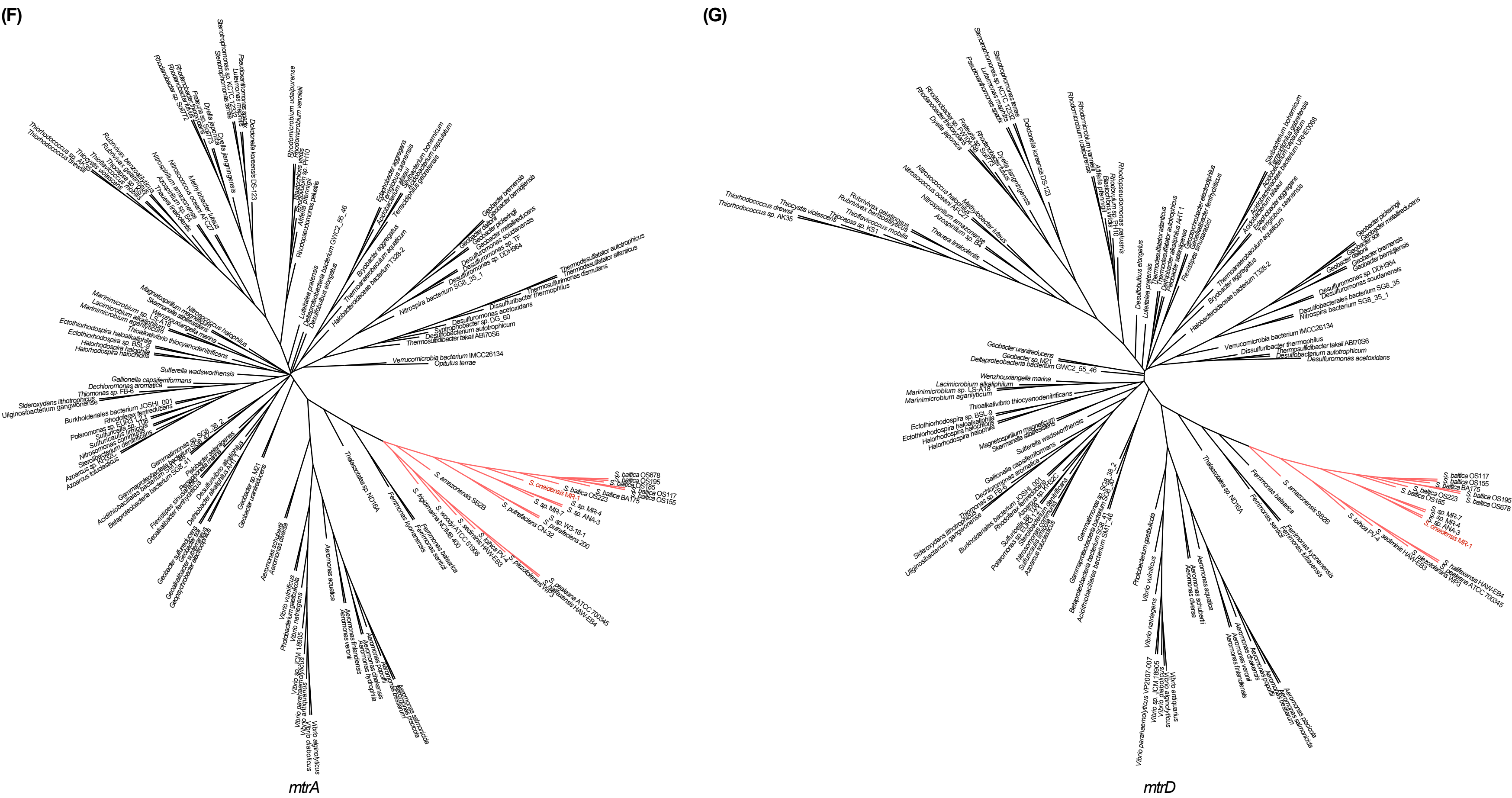

Supplement: Supplementary file 7 — Additional file 7: Figure S5. Phylogenetic relationship of mtr–omc clusters within different bacteria. The phylogenic trees built by protein sequences were showed for (A) mtrB, (B) mtrC, (C) mtrE, (D) mtrF, (E) omcA, (F) mtrA and (G) mtrD, respectively. The branches of Shewanella and clade of S. oneidensis MR-1 are marked in red. [file 13068_2018_1201_MOESM7_ESM.pdf]
